# JC Virus Encephalopathy Is Associated with a Novel Agnoprotein-Deletion JCV Variant

**DOI:** 10.1371/journal.pone.0035793

**Published:** 2012-04-19

**Authors:** Xin Dang, Christian Wüthrich, Jennifer Gordon, Hirofumi Sawa, Igor J. Koralnik

**Affiliations:** 1 Division of Neurovirology, Department of Neurology, Beth Israel Deaconess Medical Center, Boston, Massachusetts, United States of America; 2 Division of Viral Pathogenesis, Department of Medicine, Beth Israel Deaconess Medical Center, Boston, Massachusetts, United States of America; 3 Department of Neuroscience and Center for Neurovirology, Temple University School of Medicine, Philadelphia, Pennsylvania, United States of America; 4 Department of Molecular Pathobiology, Research Center for Zoonosis Control, Global COE Program for Zoonosis Control, Hokkaido University, Sapporo, Japan; University Hospital San Giovanni Battista di Torino, Italy

## Abstract

JC virus encephalopathy (JCVE) is a newly described gray matter disease of the brain caused by productive infection of cortical pyramidal neurons. We characterized the full length sequence of JCV isolated from the brain of a JCVE patient, analyzed its distribution in various compartments by PCR, and determined viral gene expression in the brain by immunohistochemistry(IHC). We identified a novel JCV variant, JCV_CPN1_, with a unique 143 bp deletion in the Agno gene encoding a truncated 10 amino acid peptide, and harboring an archetype-like regulatory region. This variant lacked one of three nuclear protein binding regions in the Agno gene. It was predominant in the brain, where it coexisted with an Agno-intact wild-type strain. Double immunostaining with anti-Agno and anti- VP1 antibodies demonstrated that the truncated JCV_CPN1_ Agno peptide was present in the majority of cortical cells productively infected with JCV. We then screened 68 DNA samples from 8 brain, 30 CSF and 30 PBMC samples of PML patients, HIV+ and HIV- control subjects. Another JCV_CPN_ strain with a different pattern of Agno-deletion was found in the CSF of an HIV+/PML patient, where it also coexisted with wild-type, Agno-intact JCV. These findings suggest that the novel tropism for cortical pyramidal neurons of JCV_CPN1_, may be associated with the Agno deletion. Productive and lytic infection of these cells, resulting in fulminant JCV encephalopathy and death may have been facilitated by the co-infection with a wild- type strain of JCV.

## Introduction

The human polyomavirus JC (JCV) [1] is the cause of widespread asymptomatic infection in healthy people [2]. Its reactivation in immunosuppressed individuals may lead to progressive multifocal leukoencephalopathy(PML), an often lethal demyelinating disease of the brain. JCV has a circular double stranded genomic DNA that includes a hypervariable non-coding regulatory region (RR) and a very stable coding region encoding the regulatory small t and large T antigens, and the capsid proteins VP1, VP2 and VP3. It also encodes the Agnoprotein, which is involved in viral DNA replication, RNA transcription and protein expression [3], [4], [5].

Although JCV predominantly infects glial cells in the CNS, a JCV-variant harboring a small deletion in the VP1 major capsid protein, demonstrated specific tropism for cerebellar granule cell neurons. Infection by this VP1-variant strain was associated with cerebellar atrophy and neurologic dysfunction named JCV granule cell neuronopathy (JCVGCN) [6], [7], [8], [9]. Recently, we have described a third clinical entity, distinct from PML and JCVGCN, JCV encephalopathy (JCVE). This disease, described in an HIV-negative patient with history of lung cancer treated with chemotherapy, is caused by a productive JCV infection of cortical pyramidal neurons. This patient presented with lesions restricted to the hemispheric gray matter on MRI, developed aphasia and progressive cognitive dysfunction and passed away 4^1/2^ month later [10].

We therefore investigated whether changes in JCV sequence could be associated with the specific tropism to cortical pyramidal neurons in this patient.

## Results

### JCV_CPN1_ has a deletion in the Agno gene and an archetype-like regulatory region

**Figure 1:**
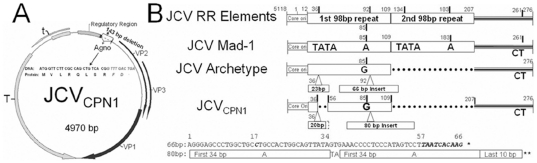
Genomic map and organization of regulatory region of JCV_CPN1_. A. The genomic map of JCV_CPN1_ shows a 143 bp deletion in the Agno gene (nt 300–442), yielding a truncated 10 aa Agno peptide, the last two aa being mutated. B. Alignment of the regulatory region (RR) of JCV_CPN1_ with the RR of prototype JCV Mad-1, usually found in CNS of PML patients and archetype JCV RR, most commonly found in urine of healthy and immunosuppressed patients alike. JCV_CPN1_ has an archetype-like RR structure with a 19 bp deletion in the 98 bp element, a 3 nt deletion in the 23 bp insert and a complex mutation in the 66 bp insert, yielding a 80 bp insert. The whole nt sequence of the archetype 66 bp insert (*) and corresponding changes in the 80 bp insert of JCV_CPN1_ RR (**) are indicated.

The full length genome of JCV infecting cortical pyramidal neurons(JCV_CPN1_, GenBank accession number JQ823124 or BankIt1524974) was amplified from DNA extracted from the fresh frozen right parietal lobe of the HIV-negative patient with JCV encephalopathy(JCVE) [10]. The major mutation found in this JCV strain is a 143 bp deletion starting from nt position 300 to 442 of the Agno gene, leaving a putative truncated Agnoprotein of 10 aa, with mutations in the last two aa (Fig. 1A). The regulatory region(RR) of JCV_CPN1_ is an archetype-like RR or type II-S [11]. Compared to the archetype RR, JCV_CPN1_ RR has a 18 nt deletion in the 98 bp element from nt 36–56, a 2 nt deletion in the 23 pb insert and an 80 bp insert in place of the 63 bp insert present in the archetype. This 80 bp insert consist of two repeats of the first 34 bp of the 66 bp insert linked by nt TA and followed by the last 10 bp of the 66 bp insert (Fig. 1B). This finding is remarkable, since archetype or archetype-like RR is usually found in JCV strains isolated from kidney or urine, and rarely from brain samples. In addition, we found a total of 22 single nt mutations scattered in the whole genome, 14 of them being silent mutations (Table 1).

**Table 1:** Single nucleotide mutations in JCV_CPN1_.

Amino acid mutations					
Position	Location	Nucleotide	mutation	aa	mutation
		Mad1	CPN1	Mad1	CPN1
1100	VP2	G	A	S	N192S
1689	VP1	A	G	N	S74N
1692	VP1	G	A	R	K75R
1850	VP1	A	G	T	A128T
1940	VP1	C	G	L	V158L
2502	VP1	A	G	K	R345K
3035	T	C	T	G	V545G
4286	T	T	C	F	S128F

Silent Mutations			
Position	Location	Nucleotide	mutations
1086	VP2	A	G
1843	VP1	G	T
2245	VP1	C	T
2260	VP1	A	T
2311	VP1	G	T
2518	VP1	G	A
2592	Inter Coding	G	T
2712	T	A	G
3131	T	T	C
3134	T	T	C
3440	T	T	C
3545	T	T	C
3599	T	G	A
4095	T	C	A
5019	Early mRNA	C	T

### Agno–deleted and Agno-intact JCV strains coexist in the brain of the patient with JCV encephalopathy

**Figure 2:**
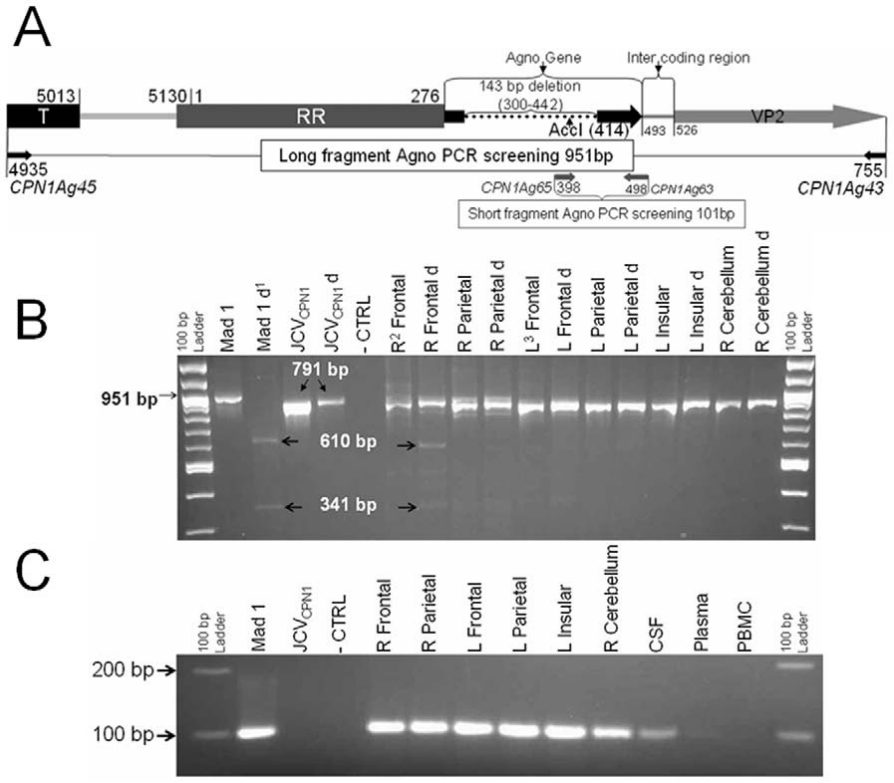
Agno gene PCR screening of brain DNA samples from the patient with JCV encephalopathy. A. Schematic representation of both the long fragment and short fragment Agno PCR screening. The long fragment Agno PCR screening was performed with primers CPN1Ag45 (4935–4958) and CPN1Ag43 (755–733), encompassing the entire Agno gene, and comprising a unique AccI restriction enzyme site. The short fragment Agno PCR screening was performed with primers CPN1Ag65 (398–420) and CPN1Ag63 (498–472), the former being located within the deleted section (300–442) of JCV_CPN1_. B. Long fragment Agno PCR screening result of Agno deletion in different brain DNA samples from JCVE patient. A 951 bp fragment was amplified from JCV Mad1 strain which contained one AccI restriction enzyme site (414), while a 791 bp fragment containing no AccI site could only be amplified from JCV_CPN1_. Two bands (341 bp and 610 bp) are visible after AccI digestion of the Mad-1 fragment (Mad-1d), while AccI digestion of the JCV_CPN1_ fragment (JCV_CPN1_ d) shows no alteration of the 791 bp PCR product. After Acc I digestion, DNA extracted from right frontal lobe showed the JCV_CPN1_ band and two digested bands coming from JCV with intact Agno gene, while all other brain samples contained the JCV_CPN1_ fragment only that could not be digested with AccI. C: Short fragment Agno PCR screening result of intact Agno gene in different brain DNA samples.A 101 bp fragment was amplified from JCV Mad-1 strains, which has an intact Agno gene, while no PCR product could be amplified from JCV_CPN1_. An intact Agno fragment could be amplified from each brain sample tested, as well as from CSF and plasma, but not from the PBMC sample from this patient. Abbreviations: d: digested by Acc I; R: right; L: left. CTRL: PCR control without DNA; PBMC: peripheral blood mononuclear cells.

Two sets of primer pairs were designed to detect Agno-deleted, JCV_CPN1_, and wild-type agno JCV strains in our clinical samples (Fig. 2A). JCV_CPN1_ was the predominant JCV strain throughout the brain. Indeed, the long fragment Agno PCR screening using primer pair CPN1Ag45 and CPN1Ag43 capable of differentiating Agno-deleted and Agno-intact JCV strains showed a strong signal of Agno-deleted JCV variant in every brain DNA sample tested. Conversely, JCV with wild-type Agno was only detected in right frontal lobe DNA sample (Fig. 2B). To avoid underestimating the presence of JCV with intact Agno due to the relatively low sensitivity of the long fragment Agno primer pair CPN1Ag45 and CPN1Ag43, we designed a more sensitive new set of primers, CPN1Ag65 and CPN1Ag63, amplifying a shorter, 101 bp fragment, that is only present in JCV strains with intact Agno gene (short fragment Agno PCR screening). This screening revealed the presence of JCV strain with intact Agno gene in every brain DNA samples of this patient (Fig. 2C). These results indicated that the predominant Agno-deleted JCV_CPN1_ strain coexisted with a minority Agno-intact JCV species in the brain of this patient.

### JCV with intact Agno gene is present in CSF and blood of the patient with JCV encephalopathy

To determine whether JCV_CPN1_ could cause systemic infection, we screened the JCVE patient's blood and CSF for the presence of Agno-deleted JCV. Long fragment Agno PCR screening remained entirely negative for both wild type and CPN type JCV strains (data not shown), while JCV with intact Agno gene JCV could be detected in the DNA extracted from plasma and CSF sample (Fig. 2C). While the lower sensitivity of the long fragment Agno PCR screening cannot allow us to formally rule out that a low amount of Agno-deleted JCV was present in blood and CSF, these results suggested that although JCV_CPN1_ was the major species in the brain of this patient, systemic infection was carried out mainly by an Agno-intact JCV strain.

### Agnoprotein deleted-JCV predominantly infects cortical gray matter of the patient with JCVE

To determine whether the Agno-deleted JCV_CPN1_ mutant was indeed responsible for the infection of cortical pyramidal neurons of the patient with JCVE, we used two Agno protein Ab to stain formalin fixed, paraffin embedded samples of this patient, and from an HIV^+^ patient with classic PML lesions as control. The first Ab, anti-Agno_1–71_ raised against 3 peptides encompassing the entire sequence of the Agnoprotein, can recognize the truncated 10 aa of JCV_CPN1_ Agno. The second Ab, anti-Agno_48–71_, detected the C terminus of the Agnoprotein, and, therefore, can recognize intact Agno protein, but cannot recognize the truncated 10 aa of JCV_CPN1_ Agno, but only intact Agnoprotein.

**Figure 3:**
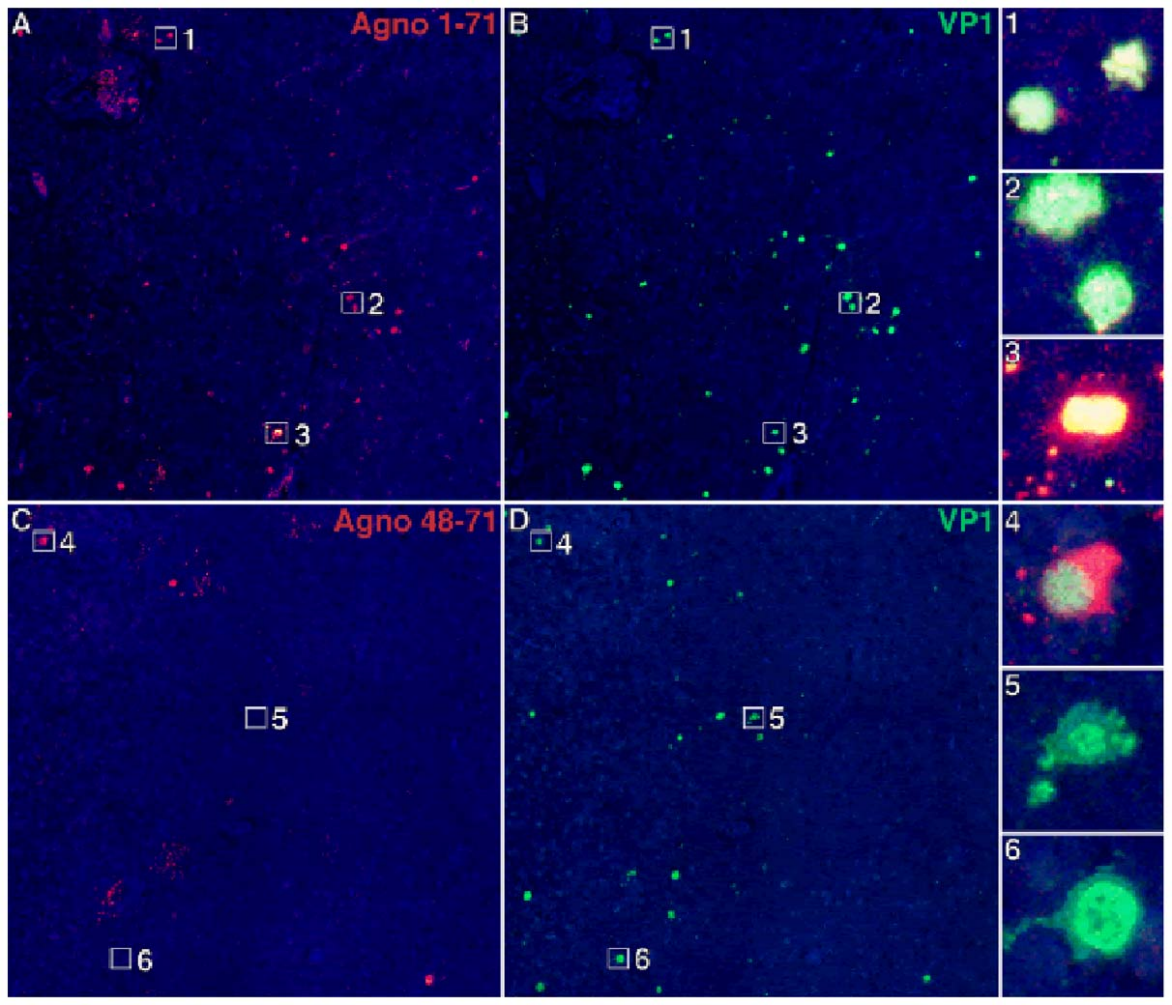
Double immunofluorescence staining of JC VP1 and Agnoprotein in JCVE patient. Double Immunofluorescence staining of JCV VP1 capsid protein and two anti JCV-Agnoprotein antibodies in cortical hemispheric brain samples of the JCV encephalopathy patient. Anti-Agno_1–71_ staining (A, red channel) colocalizes with anti-VP1 staining (B, green channel) in the patient with JCVE and double-stained cells magnified in insets # 1–3 appear yellow-white after merging of red and green channels .Conversely, anti-Agno_48–71_ shows only limited staining (C, red channel) in this patient with very rare colocalization with the anti-VP1 staining (D, green channel), magnified only in inset # 4, and the great majority of cells express VP1 only (insets #5 and 6). Scale bar: 100 µm.

**Figure 4:**
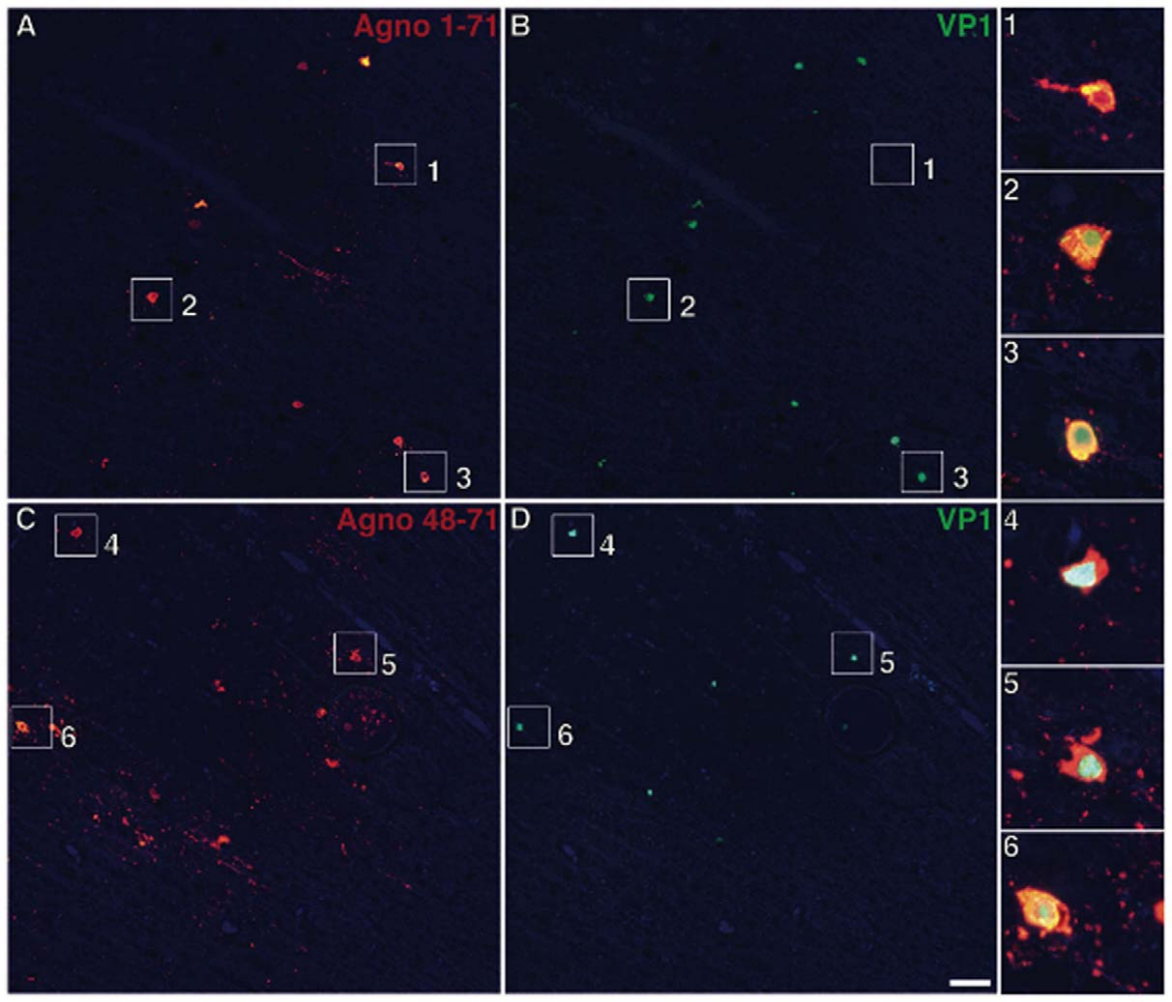
Double immunofluorescence staining of JC VP1 and Agnoprotein in classic PML patient. Double Immunofluorescence staining of JCV VP1 capsid protein and two anti JCV-Agnoprotein antibodies in brain autopsy samples of an HIV+ PML patient. Anti-Agno_1–71_ staining (A, red channel) labels the same cells as anti-VP1 staining (B, green channel) in this patient with classic PML. Similarly, anti-Agno_48–71_ staining (C, red channel) co-localizes with anti-VP1 staining (D, green channel). Magnified double-stained cells are shown in insets #1–3 and 4–6 after merging of red and green channels. Scale bar: 100 µm.

As shown in Fig. 3 A, the anti-Agno_1–71_ staining was present in all the cells also expressing the VP1 capsid protein (Fig. 3B,) in affected cortical areas of the patient with JCVE. Insets # 1–3 show the double staining of magnified cells. Conversely, most cortical cells did not stain with the anti-Agno_48–71_ Ab (Fig. 3.C) and this Ab did not colocalize with cells that also express VP1 (Fig. 3D). Based on an average of 6 different blocks of cortical areas, only 1% cells had double staining with the anti-Agno_48–71_ and VP1 Ab, magnified in inset # 4, while 99% displayed VP1 staining only (insets # 5 and 6). In classic PML lesions of an HIV^−^infected patient, however, both the anti-Agno_1–71_ and anti-Agno_48–71_ co-stained most VP1-expressing cells equally (Fig. 4). These findings suggest that the JCV_CPN1_ Agno-deletion variant was indeed the major JCV species present in the cortical pyramidal neurons of the patient with JCVE.

### Agnoprotein-expressing cells in the gray matter of the JCVE patient are neurons

**Figure 5:**
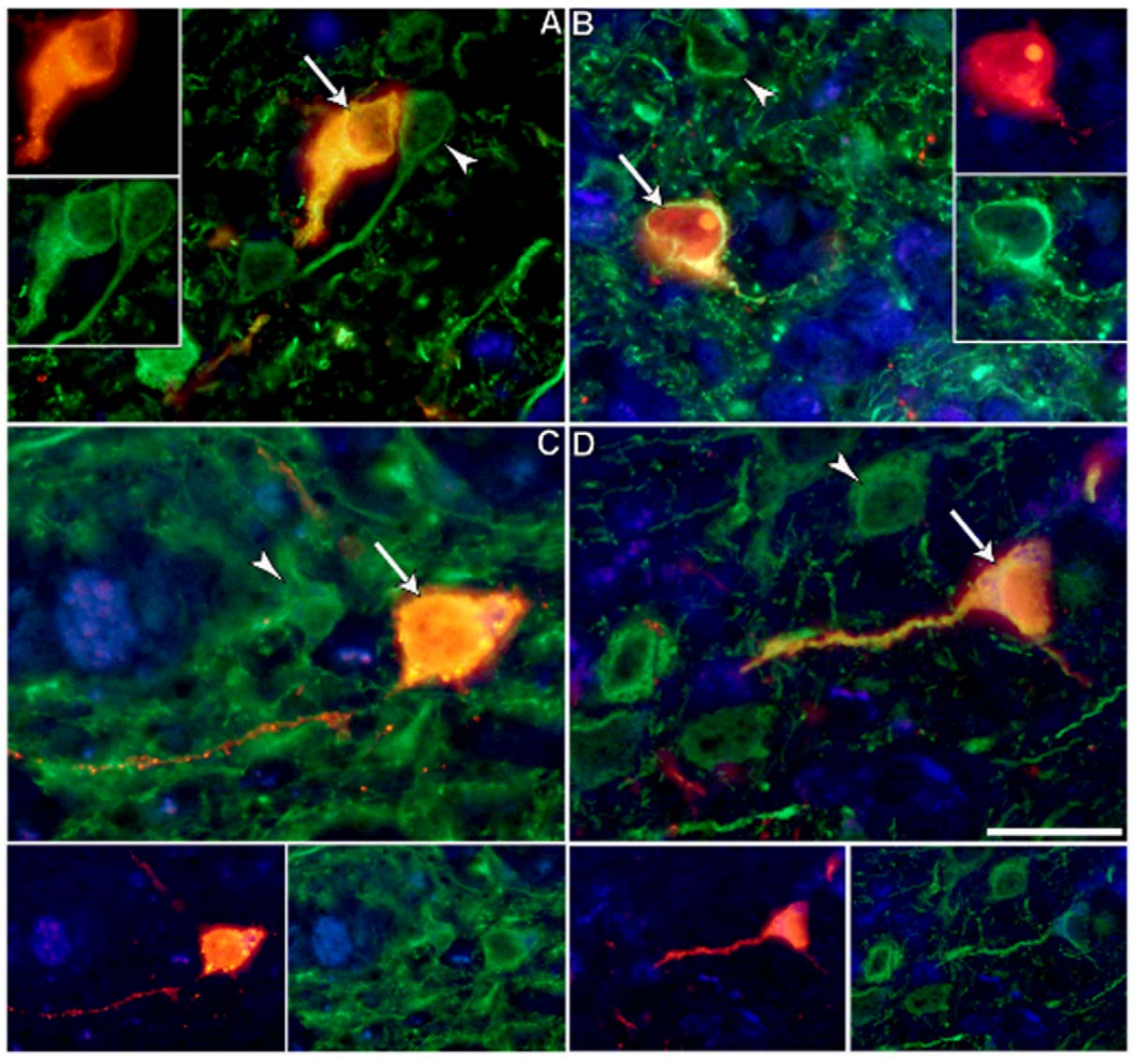
Double immunofluorescence staining of neuronal marker and JC Angoprotein in JCVE patient. Double immunostaining of Agnoprotein and a neuronal marker in the cortical hemispheric brain sample of the JCV encephalopathy patient. Many cells display both anti-Agno_1–71_ staining (red) and MAP-2 (green, panels A–D, arrows) and can be visualized in the insets with the red and green channels. These cells are interspersed among non-infected neurons (panels A–D, arrowheads) Scale bar: is 25 µm.

To determine the phenotype of agnoprotein expressing cells in the gray matter of the JCVE patient, double staining experiments were performed using the anti-Agno_1–71_ ab and the neuronal marker MAP-2. As shown in Fig. 5, numerous double stained cells-could be seen in the cortical gray matter of the JCVE patient using IFA (Fig. 5A–D). These results indicate that these cells were indeed neurons. No double-stained cells could be seen using the anti-Agno 48–71 ab and MAP-2 (data not shown), suggesting the presence of a truncated Agnoprotein in these neurons. Similarly, we did not find any Agnoprotein-expressing neurons in the case of classic PML, using either anti-Agno ab (data not shown).

### A different Agno-deleted JCV_CPN_ variant is present in the CSF of another patient with PML

**Figure 6:**
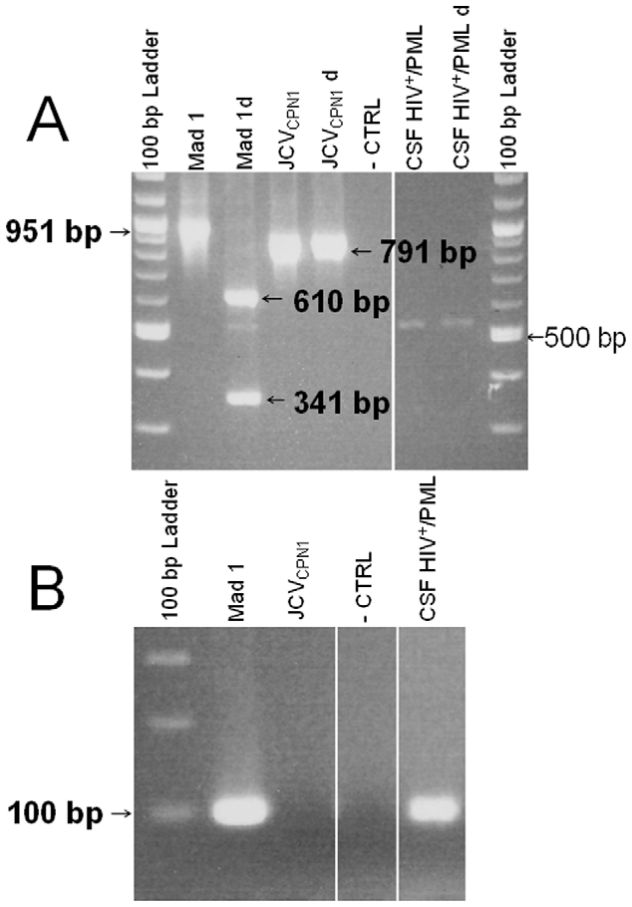
Identification of JCV_CPN2_ in other CSF samples by PCR. Agno gene PCR screening of 11 CSF DNA samples reveals another Agno deletion variant. A. Long fragment Agno PCR screening result of Agno deletion in different brain DNA samples. Compared with the 951 bp fragment amplified from Agno-intact JCV and 791 bp from JCV_CPN1_, a shorter ∼530 bp fragment was amplified from 1/11 CSF DNA samples tested, which was named JCV_CPN2_; B. Short PCR screening result showed the presence of an intact Agno JCV strain in the same CSF sample.

To determine whether JCV_CPN1_ is a unique JCV variant present only in our patient with JCVE, we screened 8 brain DNA samples(from 5 PML/HIV^+^ and 1 chronic lymphocytic leukemia/HIV^−^ patient), 30 CSF samples (20 PML/HIV^+^, 1 PML/HIV^−^, 7 HIV^+^ and 2 HIV^−^ patients), and 30 PBMC DNA samples (10 PML/HIV^+^, 10 PML/HIV^−^, 2 HIV^−^ immunosuppressed, 5 HIV^+^ and 3 HIV^−^ patients). Among all 68 clinical DNA samples isolated from 63 patients, we found one Agno-deleted JCV strain in one of the CSF samples of an HIV^+^/PML patient, which we named JCV_CPN2_ (Fig. 6A). This Agno-deleted JCV strain yielded a 530 bp amplification product by long PCR screening. Interestingly, JCV_CPN2_ also coexisted with an Agno-intact JCV strain in the CSF of this patient, as demonstrated by short fragment Agno PCR screening, which could only detect undeleted JCV strains (Fig. 6B). This sample was from a 35 year old male with AIDS and Kaposi sarcoma, CD4^+^ T cell counts of 84/µl and a plasma HIV viral load of 124,000 copies/ml, who presented with left sided ataxia and weakness. MRI showed cortical atrophy and a left cerebellar and right posterior pontine lesion consistent with PML, confirmed by positive CSF JCV PCR. Lesions stabilized on combined anti-retroviral therapy. However, the patient passed away 7 years later, from pneumonitis and sepsis unrelated to PML.

### JCV_CPN2_ consisted of two different Agno-deleted subtypes

**Figure 7:**
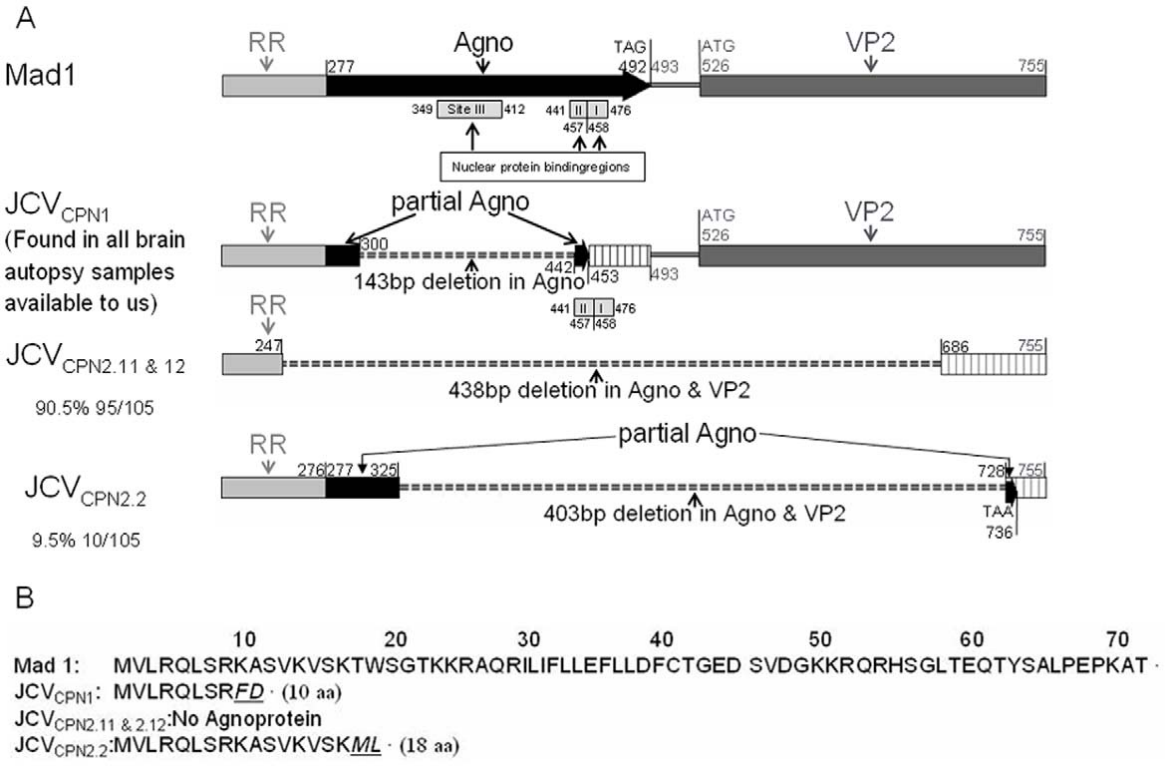
Comparison of JCV_CPN1_ and JCV_CPN2_ Agno gene organization and their final expression product. A. Alignment of the Agno gene structure of JCV prototype Mad-1, which has an intact Agno gene, JCV_CPN1_ which has a 143 bp Agno deletion and the two subtypes of JCV_CPN2_ which have a 438 and 403 bp deletion in Agno and VP2. Nuclear protein binding regions are indicated B. Alignment of the aminoacid structure of the JCV prototype Mad-1 Agnoprotein, with the 10 aa of JCV_CPN1_ and 18 aa of JCV_CPN2.2_. JCV _CPN2.1_ cannot express any Agno product.

Two different JCV subtypes were identified after long fragment Agno PCR cloning and sequencing as shown in Fig. 7A. One subtype, named JCV_CPN2.1_ which represented 90.5% of the total JCV clones, had a 438 bp deletion starting at nt 248 in the late promoter of the RR, encompassing the entire Agno gene and terminating at nt 685 in the VP2 gene (Fig. 7A). The other subtype, named JCV_CPN2.2_, which represented 9.5% clones, had a 403 bp deletion starting at nt 326 in the Agno gene, maintaining the coding potential for a mutated 18 amino acid Agno peptide, and ending at nt 728 in the VP2 gene (Fig. 7B). Both subtypes lost the initiation codon of the VP2 gene, and hence should not be able to produce a VP2 protein. The aa sequence of the undeleted Agnoprotein, the mutated 10 aa JCV_CPN1_ and 18 aa JCV_CPN2.2_ Agno deletion variants are shown in figure 7B.

### The Agno-deletion mutant JCV_CPN2_ has RR with tandem repeat pattern

**Figure 8:**
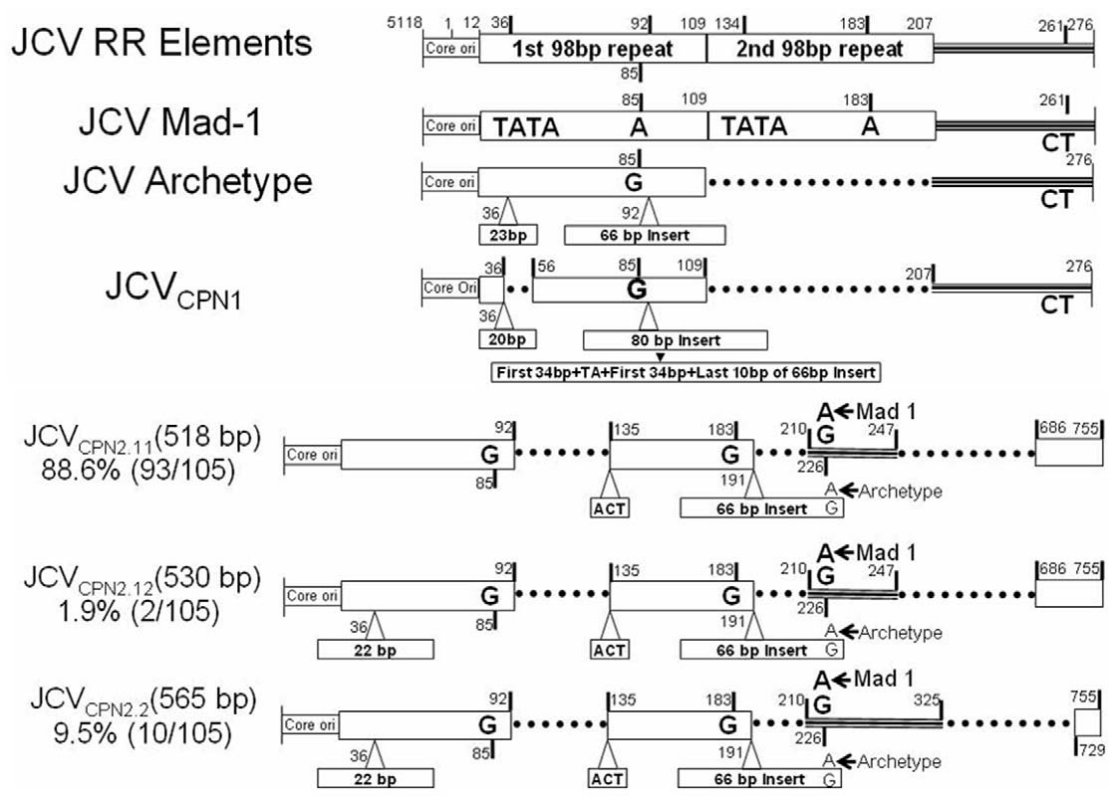
Comparison of different kinds of organization of JCV regulatory regions. Comparison of the regulatory regions (RR) of JCV prototype Mad-1, JCV archetype, JCV_CPN1_ and the JCV_CPN2_ subtypes.

To determine whether JCV_CPN2_ had an archetype-like RR similar to JCV_CPN1_, we sequenced its RR contained in the long Agno PCR fragment and compared it to JCV prototype Mad-1, archetype RR, and JCV_CPN1_ RR (Fig. 8). Compared to JCV_CPN1_, the RR had a partial duplication of the 98 bp element and truncated inserts, corresponding to a type II-R [11]. JCV_CPN2.1_ had two different types of RR. The main type called JCV_CPN2.11_, accounted for 88.6% (93/105) of the tested clones, consisted of two incomplete 98 bp elements, the second of which contained the 23 bp insert truncated to 3 bp, and an intact 66 bp insert (Fig. 8). A minority RR type, called JCV_CPN2.12_ accounting for 1.9% of clones was similar to JCV_CPN2.11_ but also had 23 bp insert truncated to 22 bp in the first 98 bp element. Finally, JCV_CPN2.2_ had a single type of RR, which was similar to JCV_CPN2.12_, but had a different deletion in the late promoter. These results indicated that Agno-deleted JCV variants may not necessarily have an archetype-like RR, and can also have RR with tandem repeat pattern, more commonly found in the CNS of patients with PML.

Nuclear protein binding regions are important for viral JCV DNA replication. Indeed the intact JCV Agno gene contains three nuclear protein binding regions. These are named region I: nt 458 to 475; II: nt 442 to 457, and III: nt 350–411 [5]. In JCV_CPN1_, binding region I was preserved, region II had a single nucleotide deletion, and region III was completely deleted. However, all subtypes of JCV_CPN2_ lost all three nuclear protein binding regions (Fig. 8).

## Discussion

This HIV-negative patient with JCVE had a unique 143 bp deletion in the JCV Agno gene, resulting in a truncation from 71 to 10 amino acids, and leading to productive infection of cortical pyramidal neurons. Although a wild-type JCV strain coexisted in all compartments, and was the only one found outside the brain parenchyma, immunostaining with two different Ab confirmed that almost all cells productively infected with JCV in the gray matter expressed the truncated agnoprotein. Since no such Agno deletion had ever been reported among more than 400 full length JCV sequences deposited in GenBank, we called this novel isolate JCV_CPN1_.

How could such an Agno-deletion mutant enter and replicate in cortical pyramidal neurons? The JCV Agno gene has been shown to have regulatory functions which impact JCV propagation *in vitro*. Indeed, three nuclear protein binding regions have been identified in the Agno gene. Whereas, the loss of Agno protein, due to mutations in the initiation codon, only resulted in a slight decrease in the JCV replication, the deletion of the entire Agno gene resulted in significant decrease of JCV replication [5]. Hence, although the protein factors which bind to the Agno gene are still unknown, there is supporting evidence that the Agno gene sequence serves as a cis-acting regulatory element in JCV DNA replication. In JCV_CPN1_, the 143 bp deletion only removed binding region III, while region I was preserved and region II had only a single nucleotide deletion. These findings may explain why JCV_CPN1_ was able to keep replicating in the cortical neurons of this patient.

Knowledge of the Agnoprotein's function is limited. This protein can be phosphorylated by protein kinase C [12], and dephosphorylated by protein phosphatase 2A [13]. Dephosphorylated Agnoprotein has been localized in the nucleus and its phosphorylated form was detected in the cytoplasm [4]. In the nucleus, Agnoprotein can bind to heterochromatin protein 1(HP1) and induce disassociation of HP1 from lamin B receptor and therefore make the nuclear envelope permissible for transportation of JCV virions [14]. In the cytoplasm, Agnoprotein can bind both fasciculation and elongation protein zeta 1 as well as microtubules and thereby promote the spread of JCV virions to uninfected adjacent cells [15]. Agnoprotein also has impact on both early and late protein expression of JCV in host cells. Deletion of the Agno gene from JCV genome resulted in decreased expression of both large T-antigen and VP1 protein [4], [5]. Therefore, the Agnoprotein plays an important role in propagation of JCV through both regulation of early and late gene transcription, and the control of virion transport. It has also been postulated that the Agnoprotein belongs to the viroporin family, which allow the exit of mature virions out of the cells [16]. Since JCV_CPN1_ does not express a fully functional Agnoprotein, this virus may therefore replicate at low levels in the nucleus of cortical pyramidal neurons, but is unable to exit these cells and spread efficiently. Because neurons do not express MHC class I proteins, the virus may escape recognition by the immune system. At some point, productive infection of neurons may have been triggered by the availability of intact Agnoprotein delivered in trans by wild-type JCV co-infection, causing cell lysis and associated neurological dysfunction. In addition, it is also possible that the mounting viral burden may have triggered local inflammation and lead to immune recognition of infected neurons.

How JCV_CPN1_ did enter cortical pyramidal neurons is unclear. Indeed, JCV has a restricted host-cell range and mainly infects glial cells in the CNS. Furthermore, this virus did not harbor the deletion in the VP1 C terminus previously detected in JCV_GCN1_, an isolate found in cerebellar granule cell neurons [6], [8]. It is therefore possible that JCV may enter a large variety of cells, and that the Agno deletion allows preferential accumulation in cortical pyramidal neurons, whereas Agno- intact strains remain mainly in glial cells. Alternatively, it is possible that a primary infection with JCV_CPN1_, occurring in this immunosuppressed individual, led to the diffuse cortical involvement observed in this patient. We have previously observed a similar histological pattern of neuronal infection in immunossuppressed monkeys inoculated with a neurotropic strain of SV40 [17]. In support of this hypothesis, this patient had anti-JCV IgG, IgM and IgA in her serum at the time of symptom onset [10]. Whether infection with Agno-intact JCV occurred simultaneously or sequentially in this patient cannot be answered by our study, because of the lack of premorbid samples. Another explanation for the neuronal tropism of JCV_CPN1_ may come from its RR. Indeed, the RR has long been identified to have a crucial role in the entry of JCV in the CNS and development of PML. However, the RR of JCV_CPN1_ has an archetype-like structure, which is usually found in urine and kidney isolates, while tandem repeat patterns are more frequent in the CNS of PML patients. Although we have recently shown that this distinction is not absolute [18], it still seems more likely that the neuronal tropism of JCV_CPN1_ was caused by its coding region rather than its RR.

The finding of another Agno-deletion mutant, JCV_CPN2_, which coexisted with an Agno-intact JCV strain in the CSF of an HIV^+^/PML patient, suggests that such variants occur in nature and have been previously overlooked. Unlike JCV_CPN1_, JCV_CPN2_ was constituted of two subtypes based on its coding region, involving deletions extending to the VP2 gene leading to a total absence of the Agnoprotein, or an Agnoprotein truncated to 18 aa, in addition to a lack of VP2 capsid protein. Unlike JCV_CPN1_, the RR of JCV_CPN2_ subtypes was of type II-R [11] with a tandem repeat pattern usually found in the CNS of PML patients, indicating that the composition of the RR is not necessarily associated with mutations of the Agno gene. This HIV^+^/PML patient had classic PML lesions as well as cortical atrophy on MRI, but no clinical evidence of JCVE. Since no cortical samples were available for histological examination, it is not possible to determine whether JCV_CPN2_ was a functionally replicating virus that could also infect neurons.

Over the past few years, the range of individuals at risk of PML has widened, and now also includes patients with autoimmune diseases treated with immunomodulatory medications. These include natalizumab for multiple sclerosis [19] and Crohn's [20] disease, efalizumab for psoriasis [21] and rituximab for rheumatologic conditions [22]. We have recently shown that JCV infection of cortical neurons occurs in up to half of the PML patients with lesions located in the gray matter of the gray-white junction [23]. Molecular studies of JC virus strains present in these areas are in progress in our laboratory. Identification of other JCVE cases will be necessary to confirm our findings. Therefore, clinicians should be aware that in addition to PML, immunocompromised patients may also be at risk for isolated gray matter lesions caused by JCV-deletion variants.

## Materials and Methods

The patients gave written consent to participate in this study according to guidelines of the Beth Israel Deaconess Medical Center (BIDMC) Committee on Clinical Investigations. The BIDMC Committee on Clinical Investigations specifically approved this study.

### DNA extraction and Quantitative PCR (QPCR) analysis of JCV viral load in brain autopsy samples

QIAamp DNA Blood Mini Kit (QIAGEN, Maryland, USA) was used to extract DNA from fresh frozen autopsy brain samples. All nucleotide (nt) positions of primers and probes correspond to the JCV prototype Mad-1 sequence. 0.5 µg brain DNA served as template in QPCR analysis. We used primer pair: JP25 (5′-CTGGTGAATTTATAGAAAGAAGTATTGCA-3′, nt 1343–1371) and JP23 (5′-GGGCCATCTTCATATGCTTCAA-3′, nt 1475–1454), and probe JCVP2Probe (6FAM-ATCTGCTCCTCAATGGATGTTGCCTTTACTT-TAMRA, nt 1392–1422 Applied Biosystems, Foster City, CA,) located in the VP2 gene. QPCR reaction was performed in duplicate by using 0.5 µg extracted brain DNA, 10 pmol of each primer, 2.5 pmol probe, 7.5 pmol reference dye (Rox), and 12.5 µl of TagMan Universal PCR Master Mix (Applied Biosystems, Foster City, CA) in a final volume of 25 µl. The cycles of amplification consisted of 2 min of 50°C, and an activation step of 10 min at 94°C, followed by 40 cycles of 94°C for 15 sec, 60°C for 1 min. QPCR was performed using 7300 Real Time PCR System (Applied Biosystems, Foster City, CA).

### Full length PCR amplification of the entire genome of JCV_CPN1_

JCV full length PCR was performed as previously described [1]. A complete JCV molecular clone was sequenced, and was named JCV cortical pyramidal neurons type 1.

### Long fragment Agno PCR screening of JCV_CPN1_ deletion in clinical samples

We used primer pair CPN1Ag45 (5′-TGTTCCCCCATGCAGACCTATCAA-3′, nt 4935–4958) and CPN1Ag43 (5′-GCCCCCGGAGCTCCAGTTATTAC-3′ nt 755–733) to detect the presence of a deletion in Agno gene in various clinical DNA samples. With this pair of primers, a long 951 bp DNA fragment can be amplified from undeleted JCV strains (such as in the JCV Mad-1 prototype), which contains one AccI restriction enzyme site at nt 414 located in the region of the Agno gene that is deleted in JCV_CPN1_ (nt 300–442 of Mad-1 strain). After digestion by AccI (New England Biolabs, Ipswich, MA, USA), the 951 bp fragment amplified from intact JCV genomes is cut in two fragments of 610 bp and 341 bp. Conversely, a 791 bp DNA fragment is amplified from the Agno deletion-mutant JCV_CPN1_, which lacks this AccI site, and therefore remains intact after AccI digestion. The cycles of amplification consisted of 10 min of 94°C pre-activation step, followed by 40 cycles of 94°C for 15 sec, 60°C for 30 sec, and 72°C for 1 min, and a final step of 72°C for 15 min.

### Short fragment Agno PCR screening of undeleted JCV strains in clinical samples

We used primer pair: CPN1Ag65 (5′-CAGGTGAAGACAGTGTAGACGGG-3′, nt 398–420) and CPN1Ag63 (5′-ACTTACCTATGTAGCTTTTGGTTCAGG-3′, nt 498–472) to amplify a short, 101 bp fragment encompassing the C terminus of the Agno gene and the N terminus of the VP2 gene. Since primer CPN1Ag65 is located in the deleted Agno sequence of JCV_CPN1_, no PCR product could be amplified from JCV_CPN1_ strain. The cycles of amplification consisted of 10 min of 94°C pre-activation step, followed by 40 cycles of 94°C for 15 sec, 60°C for 15 sec, and 72°C for 30 sec, with a final step of 72°C for 15 min.

### Immunostaining of Agnoprotein in hemispheric cortex

We used the following primary antibodies (Ab) in immunohistochemistry (IHC) experiments: Mouse monoclonal anti-VP1(PAB597) [24], Rabbit Polyclonal anti-JCV Agnoprotein Ab (a generous gift of Dr Mahmut Safak) [25] and rabbit polyclonal anti JCV Agnoprotein C terminus Ab [26]. We will refer to those two anti-Agnoprotein Ab as anti-Agno_1–71_ and anti-Agno_48–71_ respectively.

Double immunofluorescence (IF) stainings were performed with primary Abs PAB597, anti-Agno_1–71_ and anti-Agno_48–71_ Ab and anti-neuronal chicken Ab MAP-2 (Lifespan Bioscience, Seattle, WA), secondary Abs are Alexa Fluor 488 & 568 conjugated Goat anti mouse and/or rabbit secondary Ab (Invitrogen) [23].
